# Modelling multiple outcomes to improve the detection of causal mediation effects in complex intervention trials

**DOI:** 10.1186/1745-6215-12-S1-A146

**Published:** 2011-12-13

**Authors:** Neil Casey, Simon Thompson, Andrew T Prevost

**Affiliations:** 1 Department of Public Health and Primary Care, University of Cambridge, CB2 0SR, UK; 2 Medical Research Council Biostatistics Unit, Institute of Public Health, Cambridge, CB2 0SR, UK; 3 Department of Primary Care and Public Health Sciences, King’s College London, SE1 3QD, UK

## Aim

In a trial to increase physical activity in sedentary adults, there was no evidence of effect on the primary outcome, though large significant effects amongst eight related SF36 measures of general health [1]. How could such differences arise? Could they be: a true effect mediated through receiving the intervention, another systematic effect such as self-reporting bias, and/or chance? The aim was to develop reliable methods to investigate whether effects of the intervention on the SF36 outcomes were mediated through truly receiving the intervention delivered in intervention sessions.

## Methods

We adopted a structural mean modelling approach with a two-stage least-squares estimation algorithm, in order to estimate mediation effects free from confounding bias [2]. It involves predicting the mediator (number of sessions attended), and outcomes, from baseline covariates. Each individual has a personal predicted ‘counterfactual’ treatment-effect difference which is regressed on the predicted mediator using a dose-response model. Although reliably bias-free, typically these methods do not provide sufficiently precise estimates except for the simplest of models. A simulation study was designed to establish the factors driving the lack of precision. We extended the two-stage approach, using linear mixed effects and GEE modelling to enable multiple SF36 outcomes to contribute to estimation of a common mediation effect.

## Results

In this trial, attendance at sessions was invariably high, adversely affecting the precision of the estimates. From the simulation study, important factors affecting detection were identified to be the size of the trial effect and the degree to which the mediator is predictable from baseline covariates. The effect of analysing four SF36 outcomes to estimate an assumed common mediation effect was to reduce the standard error of the estimated effect by up to 40%, equivalent to offering an increase in power to detect mediation from 50% to 80%. The mediation effect was statistically significant.

## Conclusions

The extension of bias-free estimation of a mediation effect from one to multiple related outcomes offered an appreciable improvement in the power to detect mediation effects and to estimate them more precisely. The significant effect through sessions indicates that some of the effect may well be genuinely connected with receipt of intervention material. The approach requires assumptions.
